# STAT3 Expression and Its Correlation with PD-L1 Expression in Non-Hodgkin's Lymphoma and Hodgkin's Lymphoma at Dr. Saiful Anwar Regional Public Hospital in Malang, Indonesian Population

**DOI:** 10.1155/2024/7989996

**Published:** 2024-05-23

**Authors:** Ailen Oktaviana Hambalie, Eviana Norahmawati, Agustina Tri Endharti, Diah Prabawati Retnani, Nayla Rahmadiani

**Affiliations:** ^1^Department of Anatomical Pathology, Faculty of Medicine Brawijaya University, Malang, Indonesia; ^2^Anatomical Pathology Laboratory, Dr. Saiful Anwar Regional Public Hospital, Malang, Indonesia; ^3^Department of Parasitology, Faculty of Medicine Brawijaya University, Malang, Indonesia

## Abstract

**Background:**

Lymphomas are malignant lymphocyte neoplasms that globally account for 10% of cancers in individuals aged <20 years. Malignant lymphomas are divided into Hodgkin's lymphoma (HL) and non-Hodgkin's lymphoma (NHL). Despite the availability of many therapeutic modalities for lymphoma, such as *Brentuximab vedotin, Nivolumab,* and *Pembrolizumab*, it is still necessary to identify appropriate strategies with minimal side effects. Immunotherapy is a promising approach, exemplified by targeting JAK/STAT3 signaling, which can inhibit tumor growth and enhance antitumor immune responses. Hence, STAT3 (*signal transducer and activator of transcription 3*) is a promising therapeutic target. PD-L1 (*programmed death-ligand 1*), an immune checkpoint molecule, is used as a frontline treatment for various cancers. This study aims to determine STAT3 expression and its correlation with PD-L1 expression in NHL and HL to serve as a basis for further research on anti-STAT3 and its combination with other therapy targets.

**Methods:**

Samples were obtained from paraffin blocks of patients with confirmed diagnoses of NHL and HL, and then immunohistochemical staining was carried out with PD-L1 and STAT3 antibodies. The collected data were then analyzed using SPSS.

**Results:**

Among the 10 HL patients, no patients (0%) expressed STAT3, while nine patients (90%) expressed PD-L1. Among the 10 NHL patients, 1 patient (10%) expressed STAT3, while six patients (60%) expressed PD-L1. There were no significant differences in STAT3 expression and PD-L1 expression between HL patients and NHL patients. There was no correlation between STAT3 and PD-L1 expression in HL and NHL because almost all STAT3 expressions were negative.

**Conclusion:**

Although this study revealed no differences between STAT3 and PD-L1 expression in HL and NHL and no significant correlation between STAT3 and PD-L1 expression in HL and NHL, this may serve as the basis for understanding the role of STAT3 and PD-L1 in the regulation of HL and NHL, which may be useful for further research targeting STAT3 and PD-L1 immunotherapy in HL and NHL.

## 1. Introduction

Lymphomas, a heterogeneous group of malignant lymphocyte neoplasms, account for 10% of cancers in individuals aged <20 years globally and have more than 90 subtypes. Malignant lymphomas are divided into Hodgkin's lymphoma (HL) and non-Hodgkin's lymphoma (NHL). In the USA, NHL ranks as the sixth most frequent cause of cancer-related death, while globally, in 2020, 0.2% of newly reported cancer deaths were due to HL. According to GLOBOCAN 2022, the incidence in Asia for HL is 37.3% and for NHL is 42.5%. In Indonesia, Dr. Saiful Anwar Regional Public Hospital in Malang reported 408 cases of lymphoma from 2018 to 2023, consisting of 56 cases of HL and 352 cases of NHL. Lymphatic tissue, bone marrow, or extranodal sites may be involved in lymphoma, with extranodal sites being more common among NHL patients. Examples of extranodal sites include Waldeyer's ring, salivary glands, orbit, paranasal sinuses, thyroid glands, larynx, and skin [1–6].

There are many available lymphoma therapeutic modalities, and immunotherapy is a promising approach. The US Food and Drug Administration (FDA) have approved *Brentuximab vedotin, Nivolumab,* and *Pembrolizumab* as immunotherapy agents. The selection of targeted agents and optimal dosage is important, even though the overall survival (OS) of lymphoma patients has been improved by new immunochemotherapeutic regimens. In a clinical trial by Carsulo et al., durvalumab (anti-PD-L1) demonstrated an 18% overall response rate (ORR) and 8% complete response rate (CRR) in diffuse large B-cell lymphoma and a 59% ORR and 27% CRR in follicular lymphoma. However, 63% of patients experienced serious adverse events, mostly related to infection. Therefore, it is necessary to identify appropriate strategies with minimal side effects. The challenge lies in the limited immune response to immunotherapy, prompting consideration of combination therapy to improve cancer therapy outcomes [7–12].

High NF-*κ*B activity, resulting from genetic alterations in the toll-like receptor (TLR) and B-cell receptor (BCR) signaling pathways, induces the production of IL-6 and IL-10, leading to constitutive activation of JAK1 and STAT3 (signal transducer and activator of transcription 3). This activation promotes cell survival and proliferation, modulates the tumor microenvironment, promotes tumor immune evasion, and is associated with inferior clinical outcomes. Targeting JAK/STAT3 signaling can inhibit tumor growth and enhance antitumor immune responses, making STAT3 a promising therapeutic target in lymphoma. A clinical trial conducted by Li et al. regarding Napabuscin, a STAT3 inhibitor, showed potent cytotoxicity against NHL cells without significant toxicity [8, 13].

PD-L1 (programmed death-ligand 1), an immune checkpoint molecule, binds the inhibitory PD-1 receptor on T-cells and suppresses T-cell activation. PD-1/PD-L1 inhibitors have been used as frontline treatments for various cancers, such as non-small cell lung cancer, metastatic melanoma, and renal cell carcinoma. So far, the anti-PD-L1 approved by the FDA is atezolizumab, avelumab, and durvalumab. However, a clinical trial conducted by Casulo found that durvalumab-anti-PD-L1—showed a limited therapeutic effect, with more than 50% of patients experiencing side effects such as transaminitis, increased bilirubin, diarrhea, thyroid disorders, pruritus, cerebral ischemia, and gastrointestinal perforation [9, 10, 14].

This study aims to determine the differences between STAT3 and PD-L1 expression in NHL and HL, as well as the correlation between STAT3 expression and PD-L1 expression in NHL and HL. It also seeks to provide a basis for further research on anti-STAT3 and its combination with other therapy targets to improve patient outcomes.

## 2. Methods

### 2.1. Sample Collection

This study was a cross-sectional study conducted in May 2023 at the Anatomical Pathology Laboratory, Dr. Saiful Anwar Regional Public Hospital, Malang. Data were collected from paraffin blocks of patients who had a confirmed diagnosis of NHL and HL from January 2018 until May 2023.

The sample size was calculated with the following formula [15]:

(1) $$\begin{array}{c} n={\left(\frac{Z_{\alpha }+Z_{\beta }}{0,5\text{ Ln }\left(1+r/1-r\right)}\right)}^{2}+3 \end{array}$$

(2) $$\begin{array}{c} ={\left(\frac{1,96+0,84}{0,5\text{ Ln }\left(1+0,8/1-0,8\right)}\right)}^{2}+3 \end{array}$$

Description:

   *n*: Minimum sample size
 
*Z*_*α*_: Alfa standard deviation = 1.96
 
*Z*_*β*_: Alfa standard deviation = 0.84
 
*r*: minimum correlation coefficient that is considered significant = 0.8

The sampling method used was simple random sampling from patient populations that met the inclusion and exclusion criteria. The inclusion criteria included NHL and HL paraffin blocks from biopsy samples confirmed histopathologically and/or immunohistochemically. The exclusion criteria included lost or damaged paraffin blocks or when the remaining tissue was insufficient for further examination in this study.

### 2.2. Immunohistochemistry Staining and Evaluation of PD-L1 Expression

PD-L1 IHC 22C3 pharmDx was used for immunohistochemical (IHC) staining, with tonsil tissue as the control tissue. Shortly, rehydrated deparaffinized sections were subjected to the heat antigen retrieval technique. PD-L1 expression was evaluated using the Tumor Proportion Score (TPS). TPS represents the percentage of viable tumor cells showing partial or complete membrane staining (≥1+) relative to all viable tumor cells present in the sample (positive and negative). PD-L1 staining was considered negative if TPS <1%, positive if TPS ≥1%, and high PD-L1 expression if TPS ≥50%. Intensity of staining (+1 = weak, +2 = moderate, and +3 = strong staining) was also recorded [16, 17].

### 2.3. Immunohistochemistry Staining and Evaluation of STAT3 Expression

STAT3 antibody (21E7) GeneTex was used for immunohistochemical (IHC) staining, with skin tissue as the control tissue. Shortly, rehydrated deparaffinized sections were subjected to the heat antigen retrieval technique. STAT3 expression was evaluated from 5 high-power fields. STAT3 staining was considered negative if nuclear staining was <10% of tumor cells, low positive if nuclear staining was 10–50% of tumor cells, and high STAT3 expression if nuclear staining was ≥50% of tumor cells [18, 19].

### 2.4. Statistical Analysis

The normality of the data was assessed using the Shapiro–Wilk test, and because the results were not normally distributed, nonparametric tests were used. The comparison of PD-L1 and STAT3 immunoexpression between NHL and HL was assessed using the Mann–Whitney test. The correlations between immunoexpression of PD-L1 and STAT3 were assessed using Spearman correlation. All analyses were conducted using Statistical Package for the Social Sciences (SPSS) software v20 [20].

## 3. Results

### 3.1. Patients' Characteristics

Table 1 summarizes the characteristics of twenty patients with Hodgkin's lymphoma and non-Hodgkin's lymphoma based on gender and age group. Among the 10 Hodgkin's lymphoma patients, 8 (80%) were male, with most cases occurring in the 11–20 years (30%) and 21–30 years (30%) age groups. Among the 10 non-Hodgkin's lymphoma patients, 6 (60%) were male, with most cases occurring in the 41–50 years (40%) and 51–60 years (40%) age groups. There was no significant difference in gender between patients diagnosed with HL and NHL (*p* > 0.05). However, there was a significant difference in the age of patients diagnosed with HL and NHL (*p* < 0.05), with HL cases tending to occur in younger patients aged 11–30 years and NHL cases tending to occur in older patients aged 41–60 years.

## 4. STAT3 Expression in HL and NHL

Table 2 summarizes STAT3 expression in twenty patients with Hodgkin's lymphoma and non-Hodgkin's lymphoma. Among the 10 Hodgkin's lymphoma patients, none (0%) showed expression of STAT3, while all 10 (100%) exhibited no expression of STAT3. Among the 10 non-Hodgkin's lymphoma patients, none (0%) had high expression of STAT3, 1 patient (10%) had low expression of STAT3, and 9 patients (90%) showed no expression of STAT3. The Mann–Whitney test revealed no significant difference in STAT3 expression between HL and NHL (*p* > 0.05).

## 5. PD-L1 Expression in HL and NHL

Table 2 summarizes PD-L1 expression in twenty patients with Hodgkin's lymphoma and non-Hodgkin's lymphoma. Among the 10 Hodgkin's lymphoma patients, 7 patients (70%) exhibited high expression of PD-L1, 2 patients (20%) showed low expression of PD-L1, and 1 patient (10%) had no expression of PD-L1. Among the 10 non-Hodgkin's lymphoma patients, 3 patients (30%) had high expression of PD-L1, 3 patients (30%) showed low expression of PD-L1, and 4 patients (40%) exhibited no expression of PD-L1. The statistical analysis using the Mann–Whitney test revealed no significant difference in PD-L1 expression between HL and NHL (*p* > 0.05).

**Figure 1:**
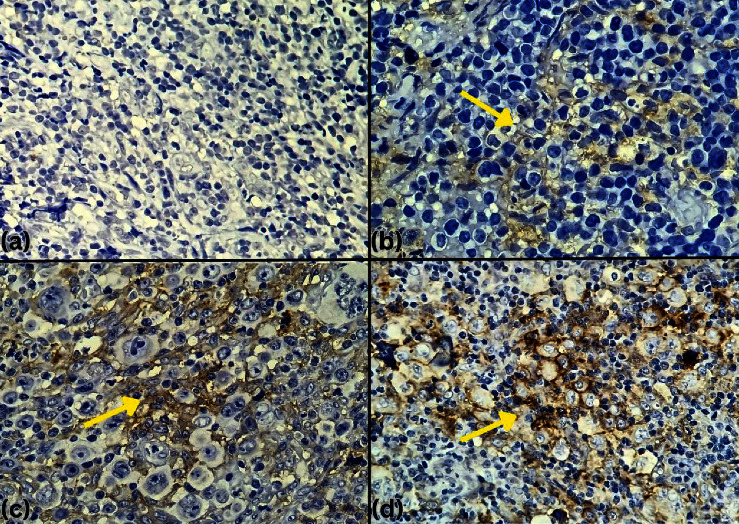
Positive immunoexpressions of PD-L1 observed under a light microscope (400× magnification). PD-L1 exhibited membrane staining with varying intensity levels: (a) no staining; (b) weak (+1); (c) moderate (+2); (d) strong (+3).

Tables 3 and 4 summarize PD-L1 staining intensity in twenty patients with Hodgkin's lymphoma and non-Hodgkin's lymphoma. Among the 9 Hodgkin's lymphoma patients with PD-L1 positivity, 1 patient (11.1%) exhibited +3 staining intensity, 7 patients (77.8%) showed +2 staining intensity, and 1 patient (11.1%) had +1 staining intensity. Among the 6 non-Hodgkin's lymphoma patients with PD-L1 positivity, 4 patients (66.7%) had +2 staining intensity, and 2 patients (33.3%) exhibited +1 staining intensity (Figure 1).

### 5.1. Correlation between STAT3 Expression and PD-L1 Expression in HL and NHL

The Spearman correlation test results between STAT3 expression and PD-L1 expression in HL patients could not be determined due to all STAT3 expressions being negative (Table 5).

**Figure 2:**
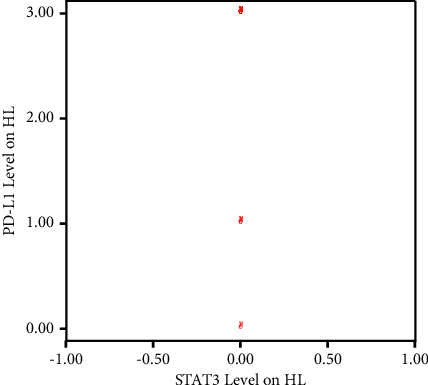
Correlation between STAT3 expression and PD-L1 expression in HL patients using SPSS v20. Since all STAT3 expressions in HL patients are negative, a linear line cannot be drawn between STAT3 and PD-L1 levels in HL patients.

Since all STAT3 expressions in HL patients were negative, a linear correlation between STAT3 and PD-L1 levels in HL patients could not be illustrated (Figure 2).

Based on the results of the Spearman correlation test between STAT3 expression and PD-L1 expression in NHL patients, a correlation coefficient value of 0.430 was obtained with a significance value of 0.214 (*p* > 0.05). Therefore, it can be concluded that there is no significant relationship between STAT3 expression and PD-L1 expression in NHL patients. In other words, high or low STAT3 expression in NHL patients is not associated with increased or decreased PD-L1 levels (Table 6).

**Figure 3:**
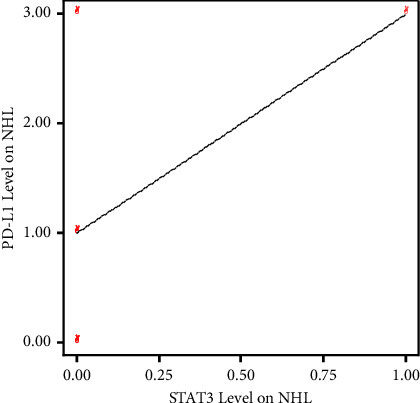
Correlation between STAT3 expression and PD-L1 expression in NHL patients using SPSS v20. Although a positive linear relationship between STAT3 expression and PD-L1 expression is observed in NHL patients, the correlation is not significant due to the small sample size.

Based on the linearity graph between STAT3 expression and PD-L1 expression in NHL patients, it appears that there is a positive linear relationship. However, due to the small sample size of NHL patients, the correlation test results did not yield significant findings (Figure 3).

## 6. Discussion

In our study, almost all STAT3 expressions are negative, which is inconsistent with findings from other studies. For instance, Seffens et al. reported that 38% of peripheral T-cell lymphoma cases showed positive STAT3 staining, while Skinnider et al. found STAT3 expression in 87% of classical HL, 46% of B-cell NHL, and 73% of T-cell NHL cases. STAT3 mediates the expression of various genes and plays a critical role in numerous cellular and biological processes, including cell proliferation, survival, differentiation, migration, angiogenesis, and inflammation. Consequently, abnormal STAT3 expression promotes malignant transformation and tumor progression through oncogenic gene expression that have been observed in numerous human cancers, and lymphoma is one of them. Moreover, STAT3 plays an important role in evading immune surveillance. Consequently, targeting JAK/STAT3 signaling can inhibit tumor growth and enhance antitumor immune responses, making STAT3 a promising therapeutic target in lymphoma [13, 21, 22]. The reason for the predominantly negative STAT3 expression in our research could be attributed to the small sample size and minimal variation among samples. Therefore, we hope that this study can serve as a foundation for further research using larger and more diverse samples to yield more significant results.

Xie et al. reported that PD-L1 is expressed in 70–87% of HL cases, and compared to HL, some subtypes of NHL exhibit lower PD-L1 expression. A study by Uccela et al. also found that PD-L1 expression is positive in more than 80% of HL cases but less than 30% of NHL cases. In certain lymphomas, such as primary mediastinal large B-cell lymphoma (PMBL), plasmablastic lymphoma, nodular sclerosis, and mixed cellularity cHL, T-cell/histiocyte-rich B-cell lymphoma, as well as virus-associated malignancies (Epstein-Barr virus-associated DLBCL and Human Herpes Virus 8-associated Primary Effusion Lymphoma), PD-L1 expression has also been reported. This discrepancy occurs because alterations of the 9p24.1 chromosome, which affect PD-L1 activation, are more frequent (61%) in HL [23–25]. This finding aligns with our study, where PD-L1 expression was observed more frequently in HL (90%) than in NHL (60%).

In our study, due to the predominantly negative STAT3 expression observed, there is no significant relationship between STAT3 expression and PD-L1 expression in HL and NHL patients. Some studies have also reported that a poorer prognosis is associated with PD-L1 or STAT3 expression. Additionally, multiple oncogenic pathways leading to the expression of PD-L1 by upregulating STAT3 expression have been documented. Exposure to inflammatory factors in the tumor microenvironment can induce PD-L1 expression, thereby facilitating immune evasion. PD-L1 protein expression in tumor cells is upregulated by interleukin (IL)-1, IL-6, tumor necrosis factor-*α* (TNF-*α*), and interferon-*γ* (IFN-*γ*). Through a STAT3-dependent mechanism, IL-6 plays an important role in the induction of PD-L1. Studies by Cheng et al. have shown that in hepatocellular cancer-associated fibroblasts, PD-L1 expression can be induced through the IL-6-STAT3 pathway, resulting in impaired T-cell function and immune suppression through the PD1/PD-L1 signaling pathway [26–29].

The limitation of this study includes the small sample size and the inherent variability associated with immunohistochemistry examination for PD-L1. Various factors may influence the results, such as tumor type, tissue sampling and preparation methods, duration of paraffin block storage, the sensitivity of the clone used, differences among manufacturers, protocols employed, and scoring methods. Additionally, gene expression may not always correlate perfectly with protein expression [30–33]. To address these limitations, future research should focus on specific tumor subtypes, utilize the recommended PD-L1 clone 22C3, and include paraffin blocks stored within a defined timeframe (2019–2023). Further confirmatory, larger-scale studies are needed to compare gene and protein expression of PD-L1. Additionally, studies regarding the combined use of anti-STAT3 with anti-PD-L1 therapies are warranted.

## 7. Conclusions

Although this study revealed no differences in STAT3 and PD-L1 expression between HL and NHL and no significant correlation between STAT3 and PD-L1 expression in HL and NHL, this may serve as the basis for understanding the role of STAT3 and PD-L1 in the regulation of HL and NHL, which may be useful for further research targeting STAT3 and PD-L1 immunotherapy in HL and NHL.

## Figures and Tables

**Table 1:** Univariate analysis of patients' characteristics based on gender and age group.

Characteristics	Hodgkin's lymphoma		Non-Hodgkin's lymphoma		*P*-value (chi-square)
	*n*	%	*n*	%	
Sex					0.329
M	8	80.0	6	60.0	
W	2	20.0	4	40.0	
Age (years)					0.032
0–10	1	10.0	0	0.0	
11–20	3	30.0	1	10.0	
21–30	3	30.0	0	0.0	
31–40	2	20.0	0	0.0	
41–50	0	0.0	4	40.0	
51–60	1	10.0	4	40.0	
71–80	0	0.0	1	10.0	

**Table 2:** STAT3 expression in HL and NHL.

Characteristics	Hodgkin's lymphoma		Non-Hodgkin's lymphoma		Exact sig. (Mann–Whitney)
	*n*	%	*n*	%	
STAT3					0.739
Negative	10	100.0	9	90.0	
Low	0	0.0	1	10.0	
High	0	0.0	0	0.0	

**Table 3:** PD-L1 expression in HL and NHL.

Characteristics	Hodgkin's lymphoma		Non-Hodgkin's lymphoma		Exact sig. (Mann–Whitney)
	*n*	%	*n*	%	
PD-L1 level					0.089
Negative	1	10.0	4	40.0	
Low	2	20.0	3	30.0	
High	7	70.0	3	30.0	

**Table 4:** PD-L1 staining intensity in HL and NHL.

PD-L1 staining intensity	Hodgkin's lymphoma		Non-Hodgkin's lymphoma	
	*n*	%	*n*	%
+1	1	11.1	2	33.3
+2	7	77.8	4	66.7
+3	1	11.1	0	00.0

**Table 5:** Correlation between STAT3 expression and PD-L1 expression in HL.

			PD-L1 level in HL	STAT3 level in HL
Spearman's rho	PD-L1 level in HL	Correlation coefficient	1,000	
		Sig. (2-tailed)		
		*N*	10	10
	STAT3 level in HL	Correlation coefficient		
		Sig. (2-tailed)		
		*N*	10	10

**Table 6:** Correlation between STAT3 expression and PD-L1 expression in NHL.

			PD-L1 level in NHL	STAT3 level in NHL
Spearman's rho	PD-L1 level in NHL	Correlation coefficient	1,000	0.430
		Sig. (2-tailed)		0.214
		*N*	10	10
	STAT3 level in NHL	Correlation coefficient	0.430	1,000
		Sig. (2-tailed)	0.214	
		*N*	10	10

## Data Availability

The underlying data for “STAT3 Expression and Its Correlation with PD-L1 Expression in non-Hodgkin's Lymphoma and Hodgkin's Lymphoma at Dr. Saiful Anwar Regional Public Hospital in Malang, Indonesian Population” can be accessed at https://doi.org/10.6084/m9.figshare.25801537.v1. The data are available under the Creative Commons Attribution 4.0 International license (CC-BY 4.0).
